# A psychometric assessment of a new Spanish version of the pain self efficacy questionnaire (PSEQ) adapted for Spanish-speaking Mexican Americans

**DOI:** 10.1016/j.jpain.2026.106345

**Published:** 2026-06-05

**Authors:** Rosa Cobian Aguilar, Scott Roesch, Sara Gombatto, Kristen J. Wells

**Affiliations:** a SDSU/UC San Diego Joint Doctoral Program in Clinical Psychology, 6363 Alvarado Ct., Suite 102, San Diego, CA 92120, USA; b Department of Psychology, San Diego State University, 5500 Campanile Dr., Atkinson Hall Room 203, San Diego, CA 92182, USA; c Department of Physical Therapy, San Diego State University, 5500 Campanile Dr., ENS 118, San Diego, CA 92182, USA

**Keywords:** Chronic Pain, Pain Self- Efficacy, Mexican Americans, Survey Adaptation, Psychometric Evaluation

## Abstract

Chronic pain affects millions of people in the United States (U.S.), with Latinx individuals facing higher risks due to chronic illness, manual labor jobs, and limited access to healthcare and resources. Many U.S. Latinx individuals are of Mexican origin and primarily speak Spanish, highlighting the need to investigate Spanish-speaking Mexican Americans’ (SS-MAs) health experiences. Psychological factors, such as pain self-efficacy (PSE; confidence in performing tasks despite pain), play a crucial role in pain management. Higher PSE is linked to increased activity engagement, but research on SS-MAs is limited partly due to the lack of culturally and linguistically adapted pain-related measures. This cross-sectional study evaluated psychometric properties (i.e., internal consistency, structural and convergent validity) of the Pain Self Efficacy Questionnaire (PSEQ) adapted for SS-MAs. A convenience sample of 182 adult SS-MAs (56.6% female; mean age= 38.3 years) with chronic pain were recruited using Qualtrics Panels. Internal consistency was high (Cronbach’s alpha = 0.916; McDonald’s omega = 0.915), and a confirmatory factor analysis showed adequate structural validity (χ^2^ (35, N = 182) = 85.757, *p* <.001; CFI = 0.929, TLI=.909, RMSEA = 0.090, SRMR = 0.053). Significant correlations were found between PSEQ scores and pain anxiety (r = −0.22, *p* <.003), catastrophizing (r = −0.15, *p* <.048), and interference (r = −0.15, *p* <.047). Although modest in strength, correlations aligned with hypothesized directions, providing preliminary evidence of convergent validity. These findings support the PSEQ’s utility for SS-MAs, but further research is needed to confirm its clinical applicability.

**Perspective:**

This study provides preliminary evidence that a culturally and linguistically adapted Mexican Spanish version of the Pain Self Efficacy Questionnaire is reliable and valid for Spanish-speaking Mexican Americans with chronic pain. Improved measures of pain self-efficacy may enhance culturally responsive research and reduce pain-related disparities in this underserved population.

## Introduction

Chronic pain is a highly prevalent and costly condition in the United States (U.S.) that substantially impacts individuals’ health and overall well-being.^1,2^ Chronic pain is commonly defined as pain that persists for three months or longer.^3^ The risk and burden of chronic pain are shaped by various sociodemographic factors (e.g., occupation, income, access to health care, age, etc.), creating increased vulnerability for some groups. Latinx individuals, one of the largest and fastest growing racial/ethnic groups in the U.S., face increased risk of chronic pain due to the disproportionate burden of chronic illnesses,^4-6^ greater representation in manual labor occupations,^7^ and reduced access to quality health care and insurance coverage compared to non-Latinx persons.^8^

Approximately 57% of U.S. Latinx persons are of Mexican origin, and about 68% of U.S. Latinx reported speaking Spanish at home.^9^ Among Mexican Americans (MAs), chronic pain is common,^10,11^ and individuals with limited English proficiency are less likely to receive comprehensive pain treatments compared to English-speaking patients.^12^ Latinos also frequently encounter ethnic and racial bias in pain care that inadequately incorporates relevant cultural factors.^12,13^ Together, these disparities highlight the importance of studying the pain-related experiences of Spanish-speaking Mexican Americans (SS-MAs).

Self-efficacy (SE), conceptualized as individuals’ beliefs about the ability to engage in actions necessary to achieve desired outcomes, has been linked to various outcomes across depression, chronic illnesses, and pain management.^14-21^ Within pain populations, pain SE refers to one’s confidence in engaging in activities or behaviors despite experiencing pain.^22^ Higher pain SE has been associated with greater physical activity, increased weight lifting, and lower disability across chronic pain samples.^23-25^ Similar associations between SE and psychological well-being among Latinas with arthritis,^26^ and SE and exercise among Hispanic individuals, compared to non-Hispanic individuals, have been documented,^27^ highlighting its relevance within Latinx populations.

Cultural and psychological factors including language and acculturation,^10,11,28^ negative beliefs about medicines,^10,28^ experiences of discrimination,^29^ and gender role expectations,^30^ have all been linked to pain-related experiences in this population. For SS-MAs specifically, cultural values emphasizing endurance, family and work obligations, acceptance, and perseverance may influence confidence in engaging in behaviors despite pain.^30,31^ It is essential to examine pain SE and other pain-related constructs within this group’s cultural contexts.

Despite the relevance of pain SE, research among SS-MAs has been limited, in part because measures are rarely adapted for SS-MAs. There are several existing measures of pain-related SE, including the Chronic Pain Self-Efficacy Scale, Self-Efficacy Scale, Physical Self Efficacy Scale, etc.^32^ However, the Pain Self Efficacy Questionnaire (PSEQ) is one of the most commonly used and clinician-preferred measures of pain-related SE.^32,33^ It assesses confidence to engage in a wide range of behaviors despite pain including social, occupational, and recreational activities, and explicitly asks respondents to take their pain into account while responding to items.^22^ While Spanish versions of the PSEQ translated in Spain have become available,^34,35^ differences in vocabulary, grammar, and idiomatic expression across Spanish-speaking groups may limit their relevance for SS-MAs. Importantly, no Spanish version of the PSEQ specifically adapted for SS-MAs existed prior to the Mexican Spanish version described in this study. However, translation alone does not establish that a measure functions equivalently across cultural and linguistic groups, as individuals may interpret items differently and the underlying construct may not be assessed in the same way.^36^ Therefore, psychometric evaluation of translated measures, like the current Mexican Spanish version of the PSEQ, is necessary to determine whether the measure demonstrates adequate reliability and validity within the target population before it can be appropriately used in research and clinical settings.^36,37^

Given the elevated burden of chronic pain among Latinx populations, the importance of culturally adapted measurement, and the lack of a Spanish PSEQ for SS-MAs, the present study aimed to evaluate a newly developed Mexican Spanish version of the PSEQ. Specifically, the study had three aims: 1) evaluate the internal consistency, 2) assess structural validity, and 3) evaluate convergent validity with pain-related outcomes. This work provides a foundation for more culturally sensitive research and clinical assessment of pain self-efficacy among SS-MAs.

## Methods and materials

### Procedures

#### Translation and adaptation

Prior studies have described procedures used to adapt the PSEQ along with other pain-related surveys (e.g. Pain Catastrophizing Scale and Pain Anxiety Symptoms Scale-20).^38,39^ For the current study, a dual-panel approach was taken to culturally and linguistically adapt the PSEQ for SS-MAs.^38,39^ Panels were conducted in April 2019. This approach has been used for the adaptation of needs-based quality of life measures.^40^ It aims to ensure quality translation beyond what can be accomplished through a standard forward-backward translation,^40^ one of the most common survey translation approaches. The dual panel approach used was a two-part process with an “expert” panel that first translated the PSEQ into Spanish and a “community member” panel that subsequently reviewed the Spanish translation and made changes accordingly. Panelists were not considered research participants. The expert panel included seven MA-identifying individuals who were bilingual in Spanish and English and had knowledge of measurement in research/surveys, pain research, and/or experience with professional translation. The community member panel included four community members who spoke Spanish and reported being of Mexican descent. All panelists were recruited through word of mouth by research staff and their colleagues. Panelists were provided with food and beverages during the panel and $30 to thank them for their time. Two research staff members who were bilingual in Spanish and English and familiar with the dual panel process and pain-related surveys served as facilitators during both panels. Facilitators assisted with the visual presentation of the survey (e.g., displayed the working document using a projector and printed copies for each panelist) and facilitation of discussion of issues that arose. During the expert panel, each panelist took turns in reading and translating a component of the survey (e.g., instructions, survey items, survey responses) out loud while the facilitators transcribed the translation verbatim. To maintain equivalence with the original English version, cultural considerations were discussed, and adaptations were made when necessary (e.g., terms were changed to more culturally familiar terms; “tidying up” was changed to “cleaning).” Once the component was translated, the other panelists were asked to indicate whether they agreed or disagreed with the translation. Panelists who disagreed were asked to provide rationale and feedback for their disagreement. Disagreements were resolved through discussion until consensus was reached among all panelists. Facilitators then documented changes on the working document. In some cases, panelists reached consensus on the most equivalent translation, but concerns about the item remained given the inherent conceptual difficulty in both languages. These concerns were documented in preparation for the community panel. Community panelists reviewed Spanish items without access to the original English versions. Similar to the initial panel, each item was read aloud and followed by discussion to evaluate its clarity, cultural appropriateness, and language. Revisions were suggested when necessary and discussed until consensus was reached. Once the adapted Spanish translation was complete, the facilitators documented procedures and formatted the document into the final translated and adapted survey. See Table 1 for translated survey items.

### Participants

Using a cross-sectional research design, a convenience sample of 182 SS-MA participants who reported chronic pain were recruited from across the U.S. via Qualtrics Panels to complete the new Mexican Spanish PSEQ. Data were collected between July 2020 and November 2020. Qualtrics Panels is an online survey-based platform that offers participant recruitment and data collection services. Eligibility criteria were provided to Qualtrics prior to initiating recruitment efforts and included participants who: 1) self-identify as Hispanic/Latinx; 2) self-identify as Mexican or of Mexican origin; 3) experience chronic non-cancer pain for 3 months or more; 4) read and understand Spanish; 5) were at least 18 years old; and 6) provided informed consent. Individuals who experience chronic cancer-related pain were excluded given the differences in etiology, mechanisms, and clinical management between cancer pain and non-cancer chronic pain. Of note, participants were not asked to specify the region(s) in which they experienced pain within the eligibility screening survey. Using convenience sampling, participants were recruited in one of two ways: 1) Qualtrics informed online-panel-provider partner organizations of our study to notify panel members who may be eligible; or 2) individuals searched for paid online research opportunities and contacted partner organizations or Qualtrics Panels directly. Once recruited, participants were directed to complete the eligibility survey. If eligible, participants were directed to provide consent and complete a 20-minute survey in Spanish. Upon completion of the survey, participants were provided with a compensation amount agreed upon and provided by Qualtrics Panels, roughly the equivalent of $7. If ineligible, participants were redirected to the end of the survey and thanked for their time. This study was approved by the San Diego State University Institutional Review Board (IRB# HS-2020–01288). All procedures were conducted in accordance with ethical standards for research involving human subjects.

### Measures

#### Pain self efficacy questionnaire (PSEQ) - Spanish

Participants completed the newly adapted 10-item Mexican Spanish PSEQ. The PSEQ is comprised of 10 items such as “I can socialize with my friends or family members as often as I used to do despite the pain,” and “I can cope with my pain without medication.” Similar to the English PSEQ, response options range from *nada seguro/a (English: not at all confident) (0)* to *completamente seguro/a (English: completely confident) (6)*. Item responses were summed to compute a total score ranging from 0 to 60, with higher scores indicating higher pain SE. The original English PSEQ demonstrated high internal consistency,^22^ adequate test-retest reliability over a period of 3 months,^22^ structural validity of a one-factor solution, and evidence of convergent validity through positive correlations with measures of coping, increasing behaviors, controlling pain; and negative correlations with measures of depression, anxiety, sickness impact, negative pain beliefs, and medication use.^22,41^ The PSEQ has since been cross-culturally adapted into more than ten languages and shortened into briefer versions.^33^ A review synthesizing the psychometric properties of all of the versions of the PSEQ in populations with musculoskeletal disorders confirmed a unidimensional factor structure across sixteen studies. Internal consistency for the PSEQ was high, with Cronbach’s alpha values ranging from.79 to.95 across twenty studies. Among other psychometric properties (e.g., test retest reliability), convergent and divergent validity was demonstrated through significant positive correlations with quality of life measures and negative correlations with measures of disability, pain interference, anxiety, depression, and pain catastrophizing, respectively.^33^

#### Pain- and health-related items - Spanish

To assess region of pain, participants were asked how much they have been bothered by stomach pain, pain in arms, legs, and joints, neck or back pain, headaches, and widespread pain. Participants were able to select all regions of the body that apply. Response options were “not bothered at all,” “bothered a little,” or “bothered a lot” for each region. To assess pain duration and frequency, display logic was implemented for each region of pain. For each region in which participants responded that pain “bothered a little” or “bothered a lot,” additional items were displayed that asked how long that pain has been an ongoing problem and how often the pain has been an ongoing problem in the past 6 months. Additionally, participants were asked to indicate whether they have ever been diagnosed with, or received treatment for, cancer. One item taken from the MOS Short-Form General Health Survey^42^ was used to assess self-perceived general health: “In general, how would you say your health is?”. Response options were “poor,” “fair,” “good,” “very good,” and “excellent”. Participants completed the Spanish version of this item.

#### Pain catastrophizing scale (PCS) – Mexican Spanish

A Mexican Spanish version of the Pain Catastrophizing Scale (PCS), translated by Cobian Aguilar and colleagues^39^ using a dual panel approach, was used to measure pain catastrophizing. The PCS has thirteen items that inquire about the degree to which individuals with pain experience catastrophizing thoughts and feelings, from not at all (0) to all the time (4). Responses to all items were summed to calculate a single, total score that ranges from 0 to 52, with higher scores indicating higher catastrophizing tendencies. A psychometric evaluation of the newly translated PCS supported high internal consistency (Cronbach’s alpha=.95, McDonald’s omega=.95), a one (pain catastrophizing) and three (rumination, helplessness, and magnification) bifactor solution, and evidence of convergent validity.^39^

#### Pain anxiety symptom scale-20 (PASS-20) – Mexican Spanish

A validated Mexican Spanish version of the Pain Anxiety Symptom Scale-20 (PASS-20), translated by Cobian Aguilar and colleagues,^38,39^ was used to measure pain-related anxiety symptom patterns. The PASS-20 is a 20-item measure that assesses overall pain-related anxiety, as well as subtypes of pain-related anxiety symptoms, with subscales of cognitive, physiological, avoidance/escape, and fear anxiety. Response options range from “never” (0) to “always” (5). Responses to items were summed to calculate a total score ranging from 0 to 100 with higher scores indicating higher pain-related anxiety generally. Subscale scores were not calculated or used in this study. A psychometric evaluation validated the newly translated PASS-20, demonstrating adequate internal consistency (Cronbach’s alpha =.93; McDonalds’ omega=.93); a hierarchical factor structure of pain anxiety as a higher order factor and cognitive, physiological, avoidance/escape, and fear anxiety as sub-factors; and adequate incremental and convergent validity.^38^

#### PROMIS pain interference - Spanish

A Spanish version of the PROMIS Pain Interference short form was used to measure pain interference over the past 7 days.^43-45^ It is composed of 8 items that measure the degree to which pain limits individuals’ physical, mental, and social activity. Items were answered using a response scale ranging from “not at all” (1) to “very much” (5). Responses to all items were summed to calculate a total raw score that can range from 8 to 40, with higher scores indicating higher pain interference. Raw total scores were converted into T-scores using the conversion table provided in the PROMIS Pain Interference Scoring Manual.^46^ The Cronbach’s alpha coefficient for this measure was.937 in the present sample.

#### PROMIS pain intensity - Spanish

A Spanish version of the PROMIS Pain Intensity item bank was used to measure pain intensity in the past 7 days.^43-45^ It is composed of 3 items that measure severity of pain at its worst, average, and present moment. All items were answered using a response scale ranging from “no pain” (1) to “very severe pain” (5). Responses to all items were summed to calculate a total raw score that can range from 3 to 15, with higher scores indicating higher pain intensity. Raw total scores were converted into T-scores using the conversion table provided in the PROMIS Pain Intensity Scoring Manual.^46^ The Cronbach’s alpha coefficient for this measure was.670 in the present sample.

#### General anxiety disorder-7 (GAD-7)- Spanish

A U.S. Spanish version of the General Anxiety Disorder-7 (GAD-7) was used to measure anxiety.^47^ The GAD-7 is composed of 7 items that measure the severity of generalized anxiety symptoms. All items were answered using a frequency scale ranging from “Not at all” (0) to “Nearly every day” (3). All item responses were summed to calculate a total score, with a score of 5–9 indicating mild anxiety, 10–14 indicating moderate anxiety, and greater than 15 indicating severe anxiety. The Cronbach’s alpha coefficient for the GAD-7 in the present sample was.919.

#### Demographics

A demographic survey was administered to all participants to collect data on age, race, ethnicity, sex assigned at birth, gender identity, sexual orientation, marital status, education level, income, number of adults and children in household, country of birth, and years in the U.S.

### Data management and statistical analysis

Several steps were taken to ensure high data quality. First, three attention check items were implemented throughout the survey (e.g., if you are reading this statement, please select ‘*completely agree’*). Participants who responded incorrectly to any attention check item were redirected to the end of the survey and thanked for their time. Second, the study team reviewed all survey completions for consistency between eligibility and demographic survey responses, duplicate entries (e.g., sequential and identical survey completions or identical responses across surveys despite differences in response options), and completeness. Less than 10% of the data were missing; thus, a listwise deletion approach was taken to address missing data. Third, item-level descriptive analyses were conducted in search of out-of-range responses or incorrect response coding.

Using SPSS, descriptive statistics were conducted to summarize characteristics of the study sample. An initial descriptive analysis was conducted to evaluate PSEQ individual item and total score trends among this sample, including measures of central tendency, variability, skewness, and kurtosis. For **study Aim 1**, internal consistency was assessed by Cronbach’s alpha and McDonald’s omega coefficients that were calculated using SPSS. Other language versions of the PSEQ have been found to have high internal consistency (original English version α=.92,^22^ Brazilian version α=.90,^48^ Chinese version α=.93,^49^ Dutch version α=.90,^50^ Catalan Spanish version, α=.92).^34^ Given the novelty of the present adaptation, it was anticipated that the adapted Mexican Spanish PSEQ for MAs would show adequate internal consistency (α≥.70) at minimum. For **study Aim 2,** Mplus version 8^51^ was used to examine the structural validity of the new Mexican Spanish PSEQ. It was anticipated that the new Mexican Spanish PSEQ would have a one-factor structure based on prior psychometric testing done with the PSEQ in other languages.^34,48-50^ A confirmatory factor analysis (CFA) was conducted to assess the model fit of the one factor structure of PSEQ among SS-MAs, with pain SE as the single factor. CFA was selected as the analytic approach because its purpose was to test the fit of an a priori hypothesized factor structure (one factor) that is based on existing evidence. Using maximum likelihood estimation, descriptive model fit was assessed using comparative model fit index (CFI), Tucker-Lewis Index (TLI), root mean square error of approximation (RMSEA), and standardized root mean residual (SRMR). Overall model fit was considered acceptable with two out of three model fit indices meeting the recommendations of Bentler (2007).^52^ CFI and TLI values greater than.90 were considered to indicate a plausible model fit^53^ and SRMR and RMSEA values lower than.08 indicated an acceptable model fit.^53,54^ Statistical model fit was assessed using a chi square test of model fit and considered to be acceptable with a non-significant *p* value at a.05 alpha level. Factor loadings greater than or equal to.40 indicated that the item significantly loaded on that factor. For **study Aim 3,** Pearson’s correlation analyses were conducted using SPSS to assess convergent validity. For the PROMIS scales for pain intensity and interference, T-scores were used for all analyses, using *M*=50 and *SD*=10. Pearson’s correlations were run to evaluate the associations between the PSEQ and the following surveys: PCS, PASS-20, PROMIS Pain Intensity, PROMIS Pain Interference, and GAD-7. These correlations assessed the association of pain SE with pain catastrophizing, pain anxiety, pain intensity, pain interference, and generalized anxiety, respectively. To examine convergent validity and using an alpha level of.05, we expected small negative correlations between scores on the PSEQ and scores on the PCS and PROMIS Pain Intensity and Interference scales, as these relationships have not been previously examined in prior research. Given that the English PSEQ score has been moderately negatively correlated with anxiety in prior research (r = −.49),^22,55^ we expected that the PASS-20 and GAD–7 (both measures of anxiety) would be moderately negatively correlated with the PSEQ, using an alpha level of.05. Additional exploratory analyses were conducted to explore the relationships between various demographic characteristics and PSEQ scores. T-tests were conducted to compare means across sex assigned at birth and nativity. Pearson r correlation analyses were conducted using total PSEQ scores and age, number of children in the household, number of adults in the household, and number of years in the U.S., if not U.S. born. One-way analysis of variance tests were conducted to compare means of groups within marital status (recoded as: married or living with a partner, married and not living with a partner or separated from their partner, divorced or widowed, and single), education (recoded as: no high school diploma or general educational development [GED], high school diploma or GED, some college or higher), and income (recoded as: U.S. $0-$50,000, $50,001-$110,000, and $110,001 or more).

## Results

### Participant characteristics

A total of 2503 Qualtrics research panelists were invited to participate in the study from July 21, 2020, to November 4, 2020, with 2129 participants (85.1%) excluded from the study following the eligibility screening survey: 229 participants were not Hispanic/Latinx (10.8%), 970 participants did not identify as Mexican (45.6%), 312 were not between the ages of 18 and 65 years (14.7%), 446 did not have chronic pain which met the study definition (20.9%), 82 had pain that was related to cancer (3.9%), 78 indicated they could not read and understand Spanish (3.7%), and 12 prospective participants did not live in the U.S. (.06%). Forty-three individuals did not provide informed consent (1.7%), and 149 were excluded based on responses to the 3 attention-check items (6%). This left 182 participants who met study inclusion criteria, signed the informed consent, and provided adequate attention to the survey.

Most participants (56.6%) were female gender identity, married and living with a spouse (65.9%), heterosexual (83.5%), and had a mean age of 38.3 years (*SD* =9.4 years). All participants identified as Hispanic/Latinx and were of Mexican descent, with most identifying as White race (73.6%) and reporting being born in Mexico (58.8%). On average, participants had lived in the U.S. for 16.8 years (*SD:* 6.7). Participants had diverse educational backgrounds. Most held college degrees (e.g., Associate’s, Bachelor’s, and graduate degrees). Most (51.6%) participants reported an income of $50,000 (U.S.) or less. See Table 2 for all demographic characteristics.

Of the 182 participants who completed the survey, 170 (94.4%) participants reported pain in more than one region of the body. One hundred thirty-four participants (73.6%) reported being bothered by stomach pain, 147 (81.2%) by pain in arms, legs, or joints, 159 (87.4%) by neck or back pain, 142 (78.0%) by headaches, and 134 (73.6%) by generalized pain or pain throughout the body. Pain duration varied widely across regions, with neck or back pain showing greater representation in the longest duration categories (1–4 years and 5 years or more) compared to other regions. See Table 3 for pain duration by region.

Examination of the skewness and kurtosis of the sample indicated that PSEQ data were normally distributed. Item-level descriptive statistics for all PSEQ items are provided in Table 4. The mean score on the PSEQ for this sample was 33.1 (*SD* = 12.4). Mean scores on the GAD-7, PCS, and PASS-20 indicated that on average participants were experiencing moderate levels of anxiety, pain catastrophizing, and pain-related anxiety. Mean T scores on the PROMIS pain intensity and interference suggest pain intensity and interference in this sample was higher than the national average (*M*= 50*, SD* = 10). Descriptive statistics for these measures are provided in Table 5.

### Study aims

**Study Aim 1)** Evaluate internal consistency. Cronbach’s alpha and McDonald’s omega coefficients were calculated for the PSEQ. Cronbach’s alpha coefficient was.916, and McDonald’s omega coefficient was.915, both indicating high internal consistency of the PSEQ. Corrected item-total correlations ranged from.602 to.778 and are provided in Table 4.

**Study Aim 2)** Evaluate structural validity. A one-factor pain SE model (Model 1) was tested using a confirmatory factor analysis. The pain SE model was indicated by all 10 items. Overall, model fit was adequate, Х^2^ [35, *N* = 182] = 85.757, *p* <.001, CFI =.929, TLI=.909, SRMR =.053. Although RMSEA was slightly elevated (RMSEA=.090), the other model fit indices met commonly recommended criteria for acceptable model fit. All standardized factor loadings were large and statistically significant, ranging from.609 to.832. Factor loadings for Model 1 are provided in Table 1.

**Study Aim 3)** Evaluate convergent validity. Pearson correlational analyses were conducted by correlating scores on the PSEQ with scores on the GAD-7, PASS-20, PCS, and PROMIS Pain Intensity and Interference Short Form scales. These analyses assessed the correlation between pain SE and generalized anxiety, pain-related anxiety, pain catastrophizing, pain intensity, and pain interference, respectively. We expected that the PASS-20 and GAD–7 (both measures of anxiety) would be moderately negatively correlated with the PSEQ, using an alpha level of.05. As hypothesized, pain SE was significantly and negatively correlated with pain anxiety (PASS-20; r= −.22, *p* =.003, pain catastrophizing (PCS; r= −.15, *p* <.05), and pain interference (r= −.15, *p* <.05), but all correlations were small. Pain SE was not significantly correlated with generalized anxiety (r = −.12, *p*=.101) or pain intensity (r= −.04, *p*=.566).

Mean total PSEQ scores did not significantly differ by country of birth, sex assigned at birth, education level, relationship status, or income group. All comparisons of PSEQ scores by demographic groups are provided in Table 6. There was a small but significant negative correlation between the number of adults in the household and total PSEQ score (*r* = −.210, *p* =.005). There were no significant correlations between total PSEQ scores and age (*r* =.030, *p* =.709), number of children in the household (*r* =−.081, *p* =.284), or years in the U.S, if not U.S.-born (*r* = −.145, *p* =.152).

## Discussion

This study presents the findings of a psychometric assessment of a new Mexican Spanish version of the PSEQ adapted for SS-MAs. The PSEQ measures one’s confidence to engage in a diverse range of activities despite pain (e.g., coping without medication, doing household chores, socializing, working, gradually becoming more active). Comparable to the original English PSEQ,^22^ the Mexican Spanish PSEQ demonstrated high internal consistency with both Cronbach’s alpha and McDonald’s omega coefficients well above.9, supporting the reliability of the items in measuring pain SE. The confirmatory factor analysis provided support for the structural validity of the Mexican Spanish PSEQ. Although the chi-square test of model fit was statistically significant, this finding should be interpreted with consideration of the documented sensitivity of the chi-square test of model fit to factors like sample size, for example.^52,53,56,57^ Psychometric research emphasizes considering multiple model fit indices rather than focusing solely on the chi-square test of model fit.^52,56,57^ In the present study CFI, TLI, and SRMR indices indicated acceptable model fit and all items demonstrated strong factor loadings (above.4) on a single factor. Together, these findings suggest that the one-factor structure provides a reasonable and theoretically consistent representation of pain SE in this sample. The results of the confirmatory factor analysis in this study are consistent with those of the original PSEQ validation studies,^22^ indicating that the structural validity of this Mexican Spanish translation is comparable to the original PSEQ developed in English.

In terms of convergent validity, pain SE measured via the PSEQ was significantly and negatively related to pain-related anxiety, catastrophizing, and interference measured via the PASS-20, PCS, and PROMIS Pain Interference Short Form, respectively. In other words, higher levels of pain-related SE, or confidence to cope with pain and engage in activities, were significantly associated with lower levels of pain anxiety, pain catastrophizing, and pain interference, as expected. Although statistically significant and in the hypothesized direction, these correlations were smaller than the correlations between the original English PSEQ and measures of related constructs (e.g., anxiety, negative pain beliefs, sickness impact). These small correlations warrant caution when interpreting and provide only preliminary evidence of convergent validity, highlighting a need for further research to explore potential reasons for this discrepancy in the strength of correlations. Perhaps there are particular demographic characteristics in this sample that moderated the relationship between the PSEQ and related constructs and/or these constructs are perceived and experienced differently among SS-MAs in this sample.

Prior studies showed significant correlations between pain SE and related constructs such as pain-specific anxiety and state-trait anxiety.^22,33^ However, the weak and non- significant correlation observed between pain SE and generalized anxiety in the present sample may actually provide evidence of divergent validity, suggesting that the PSEQ captures a construct that is distinct from generalized anxiety. Pain SE was also not significantly related to pain intensity in the present sample. The weak and non-significant correlation may be attributable to a temporal mismatch between measures, as the PSEQ asks respondents to indicate their confidence in functioning despite pain at present; while the Pain Intensity scale captures pain over the last seven days *and* right now, experiences that may fluctuate independent of perceived confidence. These results may also be due to the previously mentioned cultural factors that can impact SS-MAs’ experiences of chronic pain and in turn influence response patterns on pain measures. For example, enduring pain and continuing to function (e.g., work) despite discomfort is often valued and viewed as a personal strength by MAs,^30,31^ which may have impacted both their pain-related behaviors (e.g., persisting in life activities) and their responses to PSEQ items such as “I can do some form of work, despite the pain. (“Work” includes housework, pain and unpaid work)” and the Pain Intensity scale. This cultural value of persisting in life activities, including persisting in social activities, fun activities, and work and family life, while experiencing pain may also have had a differential impact on MAs’ experiences with anxiety, and responses on PSEQ items including “I can still do many of the things I enjoy doing, such as hobbies or leisure activity, despite pain,” potentially changing the strength but not the direction of the relationship between pain SE and general anxiety. Similarly, some MAs and other Latinx persons hold cultural beliefs that life difficulties (e.g., suffering and fatalism) should be accepted without complaints,^30,31,58^ perhaps contributing to the perception that high levels of pain intensity should not be endorsed. Furthermore, if MAs continued to persist in life activities despite pain, this continued activity may eventually result in less pain intensity, as a prior systematic review has noted that sedentary behaviors are associated with higher risk for lower back pain.^59^ Thus, there could be a significant impact of culture on the pain experience among MAs, and these cultural differences may explain the differences in the strength of the correlations found between our research and prior studies.

This study is the first to develop and evaluate the psychometric properties of a new Mexican Spanish translation of the PSEQ. A rigorous linguistic and cultural adaptation approach that integrates best practices was performed to ensure construct equivalence while employing cultural sensitivity. Data were collected from a nationwide sample of SS-MAs to ensure geographic representation in our sample. Further, pain was reported across multiple body regions and spanned from less than one month to five years or more, reflecting a broad range of pain experiences. This new Mexican Spanish version of the PSEQ is essential because SE is a key psychosocial factor influencing chronic pain outcomes; and SS-MAs, among other Latinx subgroups, may be more vulnerable to developing chronic pain given the high prevalence of other chronic illness (e.g., diabetes, cardiovascular disease) and lower trends of healthcare coverage, income, and education. The disadvantages experienced across Latinx subgroups in the U.S. may create differences in the psychological processes involved in coping mechanisms and recovery such as SE, compared to non-Latinx counterparts, but pain SE has not been well studied among SS-MAs specifically. Assessing the psychometric properties of a new Mexican Spanish translation of the PSEQ for SS-MAs can add to the literature by providing an accurate assessment of pain-related SE among SS-MAs. Measuring pain SE can enhance understanding of contributing factors and associations with coping strategies and recovery outcomes. Such insights can inform the development of targeted interventions to improve pain outcomes among this population.

Although novel, the present study is not without limitations. The data were collected from a sample of SS-MAs connected to Qualtrics Panels, suggesting some degree of literacy in using technology and navigating online research participation, limiting generalizability to individuals with lower technological literacy. While there were few associations between PSEQ scores and demographic variables, most participants were female, married, identified as heterosexual, reported having a college degree, and on average were around 38 years old, introducing a self-selection bias and limiting generalizability to SS-MAs with different demographic characteristics. Similarly, fluency in other languages was not assessed among participants, and therefore we cannot assume that all participants were monolingual Spanish speakers. Differences may exist in monolingual Spanish speakers compared to bilingual individuals and/or individuals whose Spanish has mixed origins. Additionally, this study did not differentiate between specific chronic pain conditions (e.g., musculoskeletal, neuropathic) and instead relied on reports of pain by body region, which limits our ability to evaluate whether the psychometric properties observed here generalize across distinct chronic pain conditions. Finally, the present study was cross-sectional and thus did not assess the temporal stability or predictive validity of this measure.

Despite these limitations, the findings of this study hold important research and clinical implications. The Mexican Spanish PSEQ appears to be a valid and internally reliable tool to measure pain-related SE among SS-MAs. Given the role of SE in influencing behaviors, it is an important construct to measure when delivering interventions. This tool may be a valuable measurement component in interventions aimed at improving chronic pain outcomes among SS-MAs. However, to effectively address SE during interventions for SS-MAs, its role and underlying factors must first be better understood.

The differences in the strength of associations between pain SE and other constructs observed in this study, compared to those reported in other language versions of the PSEQ, highlight the need for continued psychometric validation across cultural and linguistic groups. The heterogeneity of chronic pain experiences underscores the importance of accounting for cultural, contextual, and psychological diversity among individuals at increased risk for poor health outcomes. Future studies should consider further psychometric assessment of the Mexican Spanish PSEQ in clinical populations and exploration of predictors of pain SE with larger samples of SS-MAs. Measuring pain SE among SS-MAs can help increase our understanding of its role in maintaining or improving chronic pain and the relationship of cultural experiences to that role. Expanding our knowledge of chronic pain experiences among SS-MAs is essential to reducing chronic pain disparities that disproportionately impact Latinx populations.

## Figures and Tables

**Table 1:** Factor loadings for 1-factor model of the PSEQ.

Item	Item #	Factor Loading
Puedo disfrutar las cosas, a pesar del dolor.	P1	**.61**
*I can enjoy things, despite the pain.*		
Puedo hacer la mayoria de los quehaceres del hogar (p.ej. limpiar, lavar trastes, etc.), a pesar del dolor.	P2	**.69**
*I can do most of the household chores (e.g., tidying-up, washing dishes, etc.), despite the pain.*		
Puedo socializar con mis amigos o familiares tan seguido como antes, a pesar del dolor.	P3	**.67**
*I can socialise with my friends or family members as often as I used to do, despite the pain.*		
Puedo lidiar con mi dolor en la mayoría de las situaciones.	P4	**.72**
*I can cope with my pain in most situations.*		
Puedo hacer cierto tipo de trabajo, a pesar del dolor (“trabajo” incluye trabajo fuera o dentro del hogar, pagado o no pagado).	P5	**.71**
*I can do some form of work, despite the pain (“work” includes housework, paid and unpaid work).*		
Todavía puedo hacer muchas de las cosas queue disfruto, como pasatiempos o actividades recreativas, a pesar del dolor.	P6	**.83**
*I can still do many of the things I enjoy doing, such as hobbies or leisure activity, despite pain.*		
Puedo lidiar con mi dolor sin medicamento.	P7	**.66**
*I can cope with my pain without medication.*		
Puedo lograr la mayoría de mis metas en la vida, a pesar del dolor.	P8	**.83**
*I can still accomplish most of my goals in life, despite the pain.*		
Puedo vivir un estilo de vida normal, a pesar del dolor.	P9	**.81**
*I can live a normal lifestyle, despite the pain.*		
Puedo ser más activo poco a poco, a pesar del dolor.	P10	**.75**
*I can gradually become more active, despite the pain*		

**Table 2:** Demographic characteristics of study participants (N=182).

Characteristic	*M (SD)*
Age (in years)	38.3 (9.4)
Number of children in household	1.5 (1.2)
Number of adults in household	2.1 (0.8)
Years living in US	16.8 (6.7)
	N (%)
**Household annual income (in U.S. dollars)**	
$0- $50,000	94 (51.6)
$50,001 to $110,000	63 (34.6)
$110,001 or more	25 (13.7)
**Ethnicity**	
Hispanic/Latinx	182 (100.0)
Not Hispanic/Latinx	0 (0)
**Nativity**	
United States	75 (41.2)
Mexico	107 (58.8)
**Hispanic/Latinx Heritage**	
Mexican	182 (100.0)
Honduran	1 (0.5)
Puerto Rican	1 (0.5)
Salvadorian	1 (0.5)
Spanish	2 (1.1)
Venezuelan	1 (0.5)
**Race**	
White	134 (73.6)
Black or African American	2 (1.1)
Asian	1 (0.5)
Native American or Native Alaskan	3 (1.6)
Middle Eastern or North African	0 (0)
Native Hawaiian or another Pacific Islander	2 (1.1)
Other race ethnicity, or origin	33 (18.1)
*Hispanic*	13 (7.1)
*Latino*	10 (5.5)
*Mexican American*	3 (1.6)
*Mexican*	4 (2.1)
*Mestizo/a*	3 (1.6)
**Education level completed** *	
No diploma	12 (6.6)
High school diploma, GED, or equivalent credential	60 (33)
Associate’s degree	32 (17.6)
Bachelor’s degree	35 (19.2)
Master’s degree	35 (19.2)
Doctorate degree	1 (0.5)
**Marital Status**	
Married, living with a spouse	120 (65.9)
Married, not living with a spouse	5 (2.7)
Living with a partner	19 (10.4)
Divorced	8 (4.4)
Separated	4 (2.2)
Single	26 (14.3)
**Sex (assigned at birth)**	
Male	78 (42.9)
Female	104 (57.1)
**Gender identity**	
Man	76 (41.8)
Woman	103 (56.6)
Gender queer/Non-binary	1 (0.5)
Do not wish to answer	2 (1.1)
**Sexual orientation**	
Heterosexual	152 (83.5)
Lesbian or gay	9 (4.9)
Bisexual	11 (6.0)
Another sexual orientation	
*Pansexual*	1 (0.5)
Don’t know/ Not sure	4 (2.2)
Do not wish to answer	5 (2.7)

* Missing 7 participant responses.

*Note:* Other response options were provided for sex (assigned at birth), gender identity, and sexual orientation, but not selected. GED=general educational development

**Table 3:** Duration of pain by region of the body.

	Stomach	Arms, legs, or joint	Neck or back pain	Headaches	Generalized pain or pain throughout the body
<1 month	27 (20.1)	8 (5.4)	6 (3.8)	17 (12.0)	21 (15.7)
1–3 months	21 (15.7)	21 (14.3)	18 (11.3)	19 (13.4)	16 (11.9)
4–6 months	24 (17.9)	34 (23.1)	37 (23.3)	25 (17.6)	22 (16.4)
7–11 months	21 (15.7)	25 (17.0)	30 (18.9)	32 (22.5)	24 (17.9)
1–4 years	29 (21.6)	36 (24.5)	41 (25.8)	30 (21.1)	30 (22.4)
5 years or more	12 (9)	23 (15.6)	27 (17.0)	19 (13.4)	21 (15.7)
Total	134 (100)	147 (100)	159 (100)	142 (100)	134 (100)

*Note.* Values are presented as *n* (%). Participants were permitted to select all regions that applied.

**Table 4:** Descriptive statistics for the spanish pain self efficacy questionnaire (PSEQ) items.

Item	Mean	Standard deviation	Range	Skewness	Standard error	Kurtosis	Standard error	Corrected Item-total Correlation
P1	3.07	1.64	0–6	−.05	.18	−.44	.36	.60
P2	3.25	1.75	0–6	−.27	.18	−.76	.36	.68
P3	3.22	1.71	0–6	−.02	.18	−.98	.36	.65
P4	3.54	1.46	0–6	−.13	.18	−.50	.36	.70
P5	3.42	1.57	0–6	−.14	.18	−.58	.36	.69
P6	3.50	1.55	0–6	−.33	.18	−.25	.36	.78
P7	2.81	1.89	0–6	−.06	.18	−1.12	.36	.63
P8	3.39	1.62	0–6	−.27	.18	−.59	.36	.77
P9	3.35	1.62	0–6	−.39	.18	−.41	.36	.75
P10	3.36	1.65	0–6	−.37	.18	−.43	.36	.71

**Table 5:** Means and standard deviations of scores on study measures.

Measure	Mean	Standard Deviation	Range
PSEQ	33.07	12.41	0–60
PASS-20	62.53	21.23	6–96
PCS	32.63	11.55	3–52
GAD-7	10.65	5.81	0–21
PROMIS Pain INTEN-SF (T scores)	64.86	7.29	43.10–81.80
PROMIS Pain INTER-SF (T scores)	63.68	5.52	47.9–77.0
PROMIS Pain INTEN-SF*	9.95	2.16	4–15
PROMIS Pain INTER-SF*	27.04	7.27	9–40

*Note.* PSEQ=Pain Self Efficacy Questionnaire; PASS-20= Pain Anxiety Symptoms Scale; PCS= Pain Catastrophizing Scale; GAD-7= Generalized Anxiety Disorder-7; PROMIS Pain INTEN-SF*= PROMIS Pain Intensity Short Form; PROMIS Pain-INTER-SF*= PROMIS Pain Interference Short Form

* Raw scores

**Table 6:** Comparisons of PSEQ scores by categorical demographic groups.

Demographic Characteristic	Mean (SD)	Test Statistic	p- value
**Country of Birth**		*t* (1, 177) =.26	*p* =.795
U.S.	32.8 (12.7)		
Mexico	33.3 (12.3)		
**Sex Assigned at Birth**		*t* (1, 177) = −.14	*p* =.888
Male	32.9 (11.4)		
Female:	33.2 (13.2)		
**Education level**		*F* (2, 169) = 1.45	*p* =.238
No high school diploma or GED	30.5 (12.7)		
High school diploma or GED	31.6 (13.6)		
Some college or higher	34.6 (116)		
**Relationship Status**		F (3, 175) = 1.13	*p* =.338
Married or living with a partner	32.2 (11.8)		
Married and not living with a partner or separated from their partner	37.8 (9.6)		
Divorced or widowed	32.7 (14.6)		
Single	36 (15.5)		
**Income in U.S. Dollars**		*F* (2, 176) = 1.63	*p* =.200
$0-$50,000	31.6 (13.4)		
$50,001-$110,000	35.3 (11.3)		
$110,001 or more	33.2 (10.9)		

## Data Availability

The data that has been used are confidential. Survey respondents were assured their data would remain confidential and would not be shared.
